# Thalassemia and Nanotheragnostics: Advanced Approaches for Diagnosis and Treatment

**DOI:** 10.3390/bios13040450

**Published:** 2023-04-01

**Authors:** Zahra Tariq, Muhammad Imran Qadeer, Iram Anjum, Christophe Hano, Sumaira Anjum

**Affiliations:** 1 Department of Biotechnology, Kinnaird College for Women, 92-Jail Road, Lahore 54000, Pakistan; 2 Sundas Foundation, Shadman 1, Lahore 54000, Pakistan; 3 Department of Chemical Biology, Eure & Loir Campus, University of Orleans, 28000 Chartres, France

**Keywords:** thalassemia, gene therapy, iron overload, iron chelation, nanomedicine, nanoparticles, nanocarriers, biosensors

## Abstract

Thalassemia is a monogenic autosomal recessive disorder caused by mutations, which lead to abnormal or reduced production of hemoglobin. Ineffective erythropoiesis, hemolysis, hepcidin suppression, and iron overload are common manifestations that vary according to genotypes and dictate, which diagnosis and therapeutic modalities, including transfusion therapy, iron chelation therapy, HbF induction, gene therapy, and editing, are performed. These conventional therapeutic methods have proven to be effective, yet have several disadvantages, specifically iron toxicity, associated with them; therefore, there are demands for advanced therapeutic methods. Nanotechnology-based applications, such as the use of nanoparticles and nanomedicines for theragnostic purposes have emerged that are simple, convenient, and cost-effective methods. The therapeutic potential of various nanoparticles has been explored by developing artificial hemoglobin, nano-based iron chelating agents, and nanocarriers for globin gene editing by CRISPR/Cas9. Au, Ag, carbon, graphene, silicon, porous nanoparticles, dendrimers, hydrogels, quantum dots, etc., have been used in electrochemical biosensors development for diagnosis of thalassemia, quantification of hemoglobin in these patients, and analysis of conventional iron chelating agents. This review summarizes the potential of nanotechnology in the development of various theragnostic approaches to determine thalassemia-causing gene mutations using various nano-based biosensors along with the employment of efficacious nano-based therapeutic procedures, in contrast to conventional therapies.

## 1. Hallmarks of Thalassemia

Thalassemia syndromes can be categorized as monogenic autosomal recessive inherited hemoglobinopathies involving decreased or abnormal production of hemoglobin [1,2,3,4,5]. These are quantitative defects of hemoglobin that may be characterized by ineffective erythropoiesis, chronic hemolysis, anemia, and iron overload [6,7,8]. They may be classified as one of the commonly occurring recessive anomalies, with almost 5% of the global population being carriers of at least one globin gene mutation [1,2]. The globin genes encode globin chains that carry out the synthesis of hemoglobin. This oxygen-binding protein is a tetramer consisting of four polypeptide globin chains, including two alpha and two beta globin chains each [9,10]. Defects in alpha-like or beta-like globin chains may result in either the development of alpha- or beta-thalassemia [5]. Depending upon the type of genetic makeup, patients with thalassemia may develop variable clinical manifestations, with some being asymptomatic, while others have severe anemia and life-threatening multiple organ system complications [2,11].

Being characterized as functional hemoglobinopathy, the molecular defects and the abnormal production of the varying globin chains means thalassemia can be broadly categorized into alpha or beta [1,5,12,13]. Although thalassemia traits may exist by co-inheritance with various structural hemoglobin variants, including hemoglobin S, C, and E, the hallmark of thalassemia is an imbalance in the α/β-globin chain ratio [1,12].

The downregulation or the abnormal production of alpha-globin chains causes α-thalassemia, which can be characterized by the abnormal production of fetal (HbF) or adult hemoglobin (HbA) [14,15,16]. The α-globin gene cluster in humans is present on the p-arm of chromosome 16 (16p13.3), relatively near to the telomere (~150 kilobases) and contains paired alleles (α α/α α) [2,17,18]. Approximately 120 mutant α–globin gene variants have been investigated and found to be significantly involved in the development of the disorder [17]. Deletions in one or more genes in the alpha globin gene loci are the major cause of the development of the disease and account for about 95% of the total cases reported for alpha-thalassemia [19].

The reduction in the production of β-globin chains, because of the mutations in the β–globin gene (HBB), is responsible for the development of β -thalassemia [8,20]. The HBB gene locus is present in the short arm of chromosome 11, which also encodes the δ-globin gene, the embryonic ε-gene, the fetal γ genes, and a pseudo-gene that are involved in the production of fetal hemoglobin (HbF), hemoglobin A2, and normal adult hemoglobin (HbA) through pairing with α-globin chains [2,13]. To date, more than 250 mutations have been identified as being responsible for the occurrence of β–thalassemia, with over 200 point mutations and other larger deletions [5,8,13,17]. Genetic and molecular defects may cause abnormal gene expression at various stages, including the transcriptional and translational processes resulting in the abnormal production of β–globin chains that cause the disorder [17,19,21].

### Pathophysiological Pathways

During fetal development, the production of α-globin and γ-globin starts and continues for the initial 6 months of life. However, within one year, the γ-globin production is replaced by β-globin synthesis to produce adult hemoglobin [2,22,23]. The unbalanced production of these α-globin and β-globin chains is the main pathophysiological mechanism involved in the development of thalassemia. The decreased or abnormal synthesis of one globin chain leads to the toxic accumulation of the other globin chains, which causes a drastic reduction in functional hemoglobin [24,25,26,27]. Moreover, the excessive globin chains, after aggregating, may form reactive oxygen species and damage the red blood cell membranes, which may lead to hemolysis and abnormal erythroid maturation [3,27].

**Figure 1:**
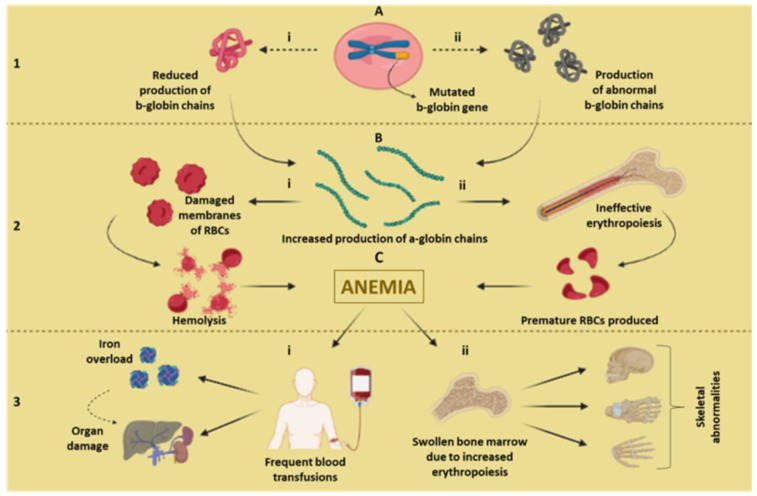
(**1A**) The beta-globin gene responsible for producing beta-globin chains of hemoglobin is mutated due to either (i) production of b-globin chains being reduced, or (ii) abnormal b-globin chains being produced. (**2B**) Abnormality of b-globin chains causes increased production of a-globin chains due to either (i) the membranes of RBCs getting damaged causing hemolysis, or (ii) RBC precursors in the bone marrow getting precipitated causing ineffective erythropoiesis, premature death, or premature production of RBCs. (**2C**) Hemolysis and ineffective erythropoiesis cause anemia. (**3**) To overcome anemia, either (i) frequent blood transfusions are required, which ultimately cause iron overload and organ damage, or (ii) vigorous signals to increase the RBC production causes the swelling of the bone marrow, which causes skeletal abnormalities.

In the case of β-thalassemia, the reduced or abnormal production of β-globin chains, due to genetic defects, causes the accumulation of α-globin chains, which on one hand, cause functional defects, and on the other, cause ineffective erythropoiesis and hemolysis through autophagy and apoptosis, which lead to severe anemia and requires frequent blood transfusions, multisystem organ defects, and bone deformities (Figure 1) [2,5,8,26,27,28].

## 2. Conventional Therapeutic Approaches

### 2.1. The Complexity of Thalassemia Detection

The complexities of thalassemia differ among different individuals. Depending upon the genetic defects and severity of the symptoms, the patients can be clinically divided into transfusion-dependent thalassemia (TDT) or non-transfusion-dependent thalassemia (NTDT) patients; the former requires frequent blood transfusions, while the latter do not need transfusion-therapy [29,30]. The diagnostic criteria involve the examination of the red blood cell indices, hemoglobin, and DNA for which automated hematology analyzers, electrophoresis, and high-performance liquid chromatography (HPLC) may primarily be used [11]. Due to the presence of numerous mutations, the diagnostic and therapeutic criteria of the type of thalassemia are complex and further complicate the treatment regimens.

### 2.2. Treatments and Complications of Thalassemia

Depending on the type of thalassemia patients, perturbations i.e., anemia, iron toxicity, metabolic defects, etc., during the disorder are managed through various conventional modalities, including blood transfusion, splenectomy, HbF induction, hydroxyurea, iron chelation therapy, usage of various drugs, and bone marrow and hematopoietic stem-cell transplantation (HSCT) [27,28,29,30,31,32,33] (Table 1). Although the life expectancy has been significantly improved through these treatments, allowing more patients to enter adulthood by increasing the survival rate, the physical, psychological, and financial burden has led the investigators to identify new simple, and effective therapeutic pathways to cater toward the complexities associated with thalassemia [31,34]. Some novel therapeutic approaches include erythropoiesis modification strategies, gene therapy, and gene editing technologies: using the editing tools like zinc-finger nucleases (ZFN), transcription activator-like effector nucleases (TALENS), and clustered regularly interspaced short palindromic repeats (CRISPR) and CRISPR-associated-nuclease 9 (CRISPR-Cas9), are under investigation [22,23,24,25,28,29,30,31,32,33,34].

## 3. Biomedical Landscape of Nanotechnology

With the advancements in clinical and biomedical research fields, novel cellular, molecular, and environmental determinants of diseases are being revealed [35]. All individuals having unique genetic makeups are susceptible to different diseases in different ways due to recombination as well as the constant influx of genetic mutations [36,37,38,39]. Different disorders, particularly thalassemia syndromes, might be caused by single nucleotide polymorphisms, copy number variations, frameshift mutations, or other structural mutations [39]. Diagnostic approaches including mutation detection methods (PCR and RFLPs), gene sequencing, or genome-wide association studies and therapeutic modalities, including pharmacological drug development, enzyme replacement therapies, oligonucleotides, gene and targeted cell therapies, and gene editing technologies are being developed for various diseases, including thalassemia [40,41,42,43,44,45,46,47]. Genetic testing may be used for the detection of various mutations; however, it requires expensive platforms and complex processes to detect small concentrations of single nucleotide variations, which is the biggest challenge among the cohorts of wildtype genes [46,47].

Nano-based approaches have recently gained a lot of popularity at being used in the healthcare system for diagnostic and therapeutic purposes for various disorders. The use of several engineered nanomaterials, with a size range of about 1–100 nm in at least one dimension, offers the possibility of their potential use in diagnostic and therapeutic purposes [48,49]. Having unique electrical, chemical, magnetic, optical, and biological properties, including non-invasive, simple, portable nature; high sensitivity, selectivity and reliability, and inexpensive diagnostic platforms, mean that nanotechnology and nanomedicine can open new avenues in biomedical science and lead to the development of novel biosensors and therapeutic approaches, specifically to cure hemoglobinopathies [46,47,48]. Keeping in view the increasing trend of precision medicine, also known as personalized medicine or customized drug production according to the genes, environment, and lifestyle of each individual, nanomedicine i.e., the use of various types of organic and inorganic nanoparticles in drug development, drug delivery, and various other therapeutic and diagnostic purposes, is under rapid investigation to develop novel and efficacious therapeutic interventions and diagnostic approaches for different diseases [42,48,49,50,51]. The manipulation of materials by nanotechnology, which may help in the alteration of physical and chemical properties, or to overcome drug-related problems including poor solubility or poor bioavailability, brings it under the spotlight for use by researchers [49,52,53].

**Figure 2:**
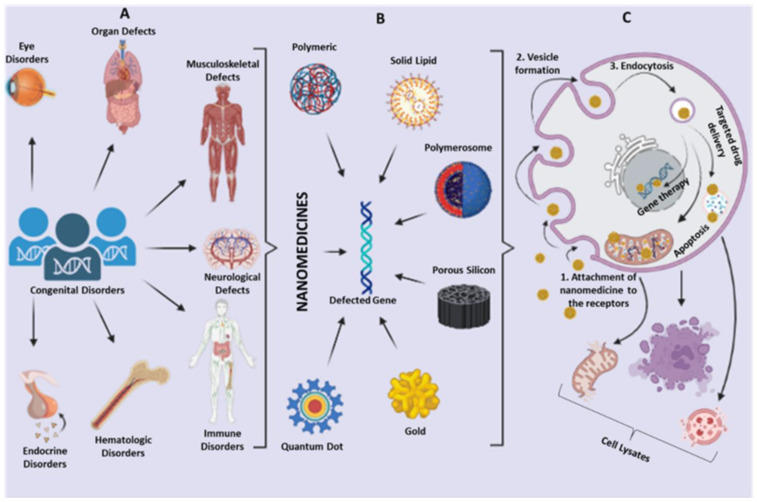
Treatment of genetic disorders through nanomedicines. (**A**) Congenital disorders of varying kinds and degrees occur, which are difficult to be diagnosed and treated. (**B**) Nanomedicines incorporating a wide variety of nanoparticles can be used for the prognosis and diagnosis of such disorders. (**C**) The targeted drug delivery as well as gene therapy, via nanomedicines, may be used for the treatment and follows the mechanism of endocytosis.

Nanoparticles have emerged as a promising tool to overcome macromolecular drug-related problems, such as low permeability through biological membranes, short biological half-life, large size, high molecular weight, and structural instability [54,55,56,57,58]. Nanoparticles vary depending on their size, structure, function, and use. Novel nanostructures, including polymeric micelles, polymeric nanoparticles, dendrimers, polymer-drug conjugates, and liposomes are classified as organic nanoparticles, whereas, carbon nanotubes, carbon nanofibers, gold nanoparticles, quantum dots, magnetic nanoparticles, nanographene, and metal-based nanoparticles have been categorized as inorganic nanoparticles [48,59,60,61,62,63,64]. These are used in the treatment and diagnosis of various genetic disorders through their use in gene therapy, cancer therapy, biomedical imaging, tissue scaffolds, implantable materials, nanodevices i.e., biosensors, and drug delivery systems (Figure 2) [49,52,64,65,66]. These advancements are being particularly explored for accurate diagnosis and treatment of thalassemia.

## 4. Nanobiosensor for the Diagnosis of Thalassemia

Nanotechnology-based methods for the detection of mutations, such as incomplete aggregation, by identifying target DNA, usage of several hybrid techniques with optical nanocarriers, or amalgamation of sensing methods with nanotechnology have been developed. Ag, Au, graphene, silica, and quantum-based nanostructures have been studied widely to demonstrate their diagnostic and therapeutic properties in various disorders, including hemoglobinopathies [47]. The potent role of nanoparticles and nanosensors in the diagnosis of anemia, blood cancer, or other bleeding disorders including thalassemia has been under investigation by researchers to develop certain effective, and cost-efficient methods with higher sensitivities [67].

Thalassemia involves qualitative and quantitative defects of hemoglobin due to how the individual suffers from anemic conditions and other complications [68]. Lipid peroxidation and reactive oxygen species formation in the patient’s body causes oxidative stress, which reduces the defense mechanisms involving antioxidants and causes damage to the cells [69]. The analysis of hemoglobin using colorimetric methods, fluorescence spectroscopy, specific gravity, Kurt electric resistance, spectrophotometry, and electrochemical methods have been developed [70]. Of these methods, electrochemical analysis is the most reliable and efficient method to be used for the quantification and detection of hemoglobin [71]. Electrochemical sensors with varying efficiencies have been developed [70]. Different nano-based diagnostic systems are given in Table 2.

### 4.1. Carbon-Based Biosensors

The principle of change in electric signals due to the selective response by the biochemical receptor has been used in the development of electrochemical biosensors. These biosensors can be used to detect the target, which in the case of thalassemia is hemoglobin [72,73,74]. Keeping in view the macromolecular structure and biomolecular alignment of the hemoglobin molecules, Xie et al. developed a Co_3_O_4_-doped carbon nanofiber (CNF) composite, which was modified by a carbon ionic liquid electrode (CILE). The hemoglobin was immobilized on it and its electrochemical behavior was investigated to detect the electrochemical, conformational, and structural alterations in diseased conditions [75]. Another unique approach was adopted by Darabi et al. to determine the vitamin C level and deferoxamine in thalassemic patients using a carbon paste-based electrode that was coupled to 1-ethyl-3-methylimidazolium chloride, as an ionic liquid (IL), and CdO-nanoparticle/rGO (IL/CdO/rGO/CPE) and was studied using a multivariate curve resolution alternating least squares (MCR-ALS) algorithm. This efficient analysis of the samples of both thalassemia major and minor patients with high accuracy demonstrated the efficacious use of carbon-based biosensors for further clinical investigations [76].

Different biosensors have been prepared using graphene and graphene derivatives because of their exceptional sensing abilities (e.g., electron transport capabilities, specific surface area, electric properties, and flexibility). These could be used for the detection of various molecular substances, such as nucleic acids, proteins, or small molecules using differential signaling approaches involving fluorescent, electrochemistry, and surface plasmon resonance [77]. Chen et al., in their experimental work, proved that a Palladium–graphene (Pd-GR) nanocomposite can be manipulated to fabricate third-generation electrochemical biosensors by fixing the Hb carbon ionic liquid electrode and modified by the Pd–GR nanocomposite [78]. Zhan et al., in another study, proved that the amalgamation of the 3D porous hybrid of the graphitic C_3_N_4_ nanoparticle with graphene, and Co_2_Al layered double hydroxide nanosheets could serve as a promising material for the production of third-generation biosensors [79]. Based on these developed biosensors, using graphene nanoparticles in hybrid technology, graphene oxide-tellurium nanowires (TeNWs/GO) have been developed for the first time by Sana et al. to quantitatively determine the hemoglobin of β-thalassemia major patients. Graphene oxide played a role in the enhancement of conductivity and surface area of the material, while tellurium nanowires improved the charge transfer mechanism. Hemoglobin from blood samples of β-thalassemia major patients was detected by this sensor, which illustrated lower levels of hemoglobin and decreased production of red blood cells in these patients [70].

### 4.2. Quantum Dot-Based Diagnosis

Quantum dots (QDs) are colloidal semiconductor nanocrystals with unique optical and electronic properties, which make them advantageous over conventional fluorophores. The fluorophore behavior is significantly influenced by the excitation width, emission wavelengths, decays, and photostability [80]. A nano-diagnostic genotyping method involving ligase reaction coupled with quantum dots and magnetic nanoparticle-based probes has been developed for the detection of point mutations in the human beta-globin gene (IVS-II-I G→A point mutation). In the presence of mutation sites, the ligase reaction proceeded with allele-specific probes. Allele-specific probes were bound by streptavidin-coated magnetic nanoparticles at one end and to a conjugate at the other end. The change in fluorescence color by the quantum dots indicated the genotypes. This non-PCR-based nano-diagnostic mutation detection method had 85.45% sensitivity and 95.77% specificity when used in the detection of thalassemic mutations in globin genes [81]. Yet, in the research conducted by Heidari et al., to detect the same beta-globin gene point mutation in thalassemic patients in Iran, the same nano-based ligation assay magnetic nanoparticles and quantum dot-labeled probes were used. Of the 50 tested DNA samples, 72% of the samples had the mutation, which confirmed the efficacious use of the nano-based mutation detection approach as having higher accuracy, sensitivity, specificity, and cost-effectiveness [82].

Recently, a CdS/TiO_2_ nanocomposite-based molecularly imprinted photo-electrochemical sensor has been developed for the detection of hemoglobin under the irradiation of visible light. A heterojunction of CdS quantum dots with TiO_2_ was formed to increase the photogenerated current of the sensor by efficacious transfer of charges under visible light. The sensor was specifically prepared for the detection of hemoglobin, keeping in view the principle of a decrease in the photocurrent, in the case of the attachment of the hemoglobin to the sensor. Depending upon the attachment patterns and fluorescence, the conformational changes and presence of the disorder could be detected through this efficient method [83].

### 4.3. Metal Nanoparticles-Based Diagnosis

#### 4.3.1. Gold Nanoparticles-Based Diagnosis

Gold nanoparticles with distinctive chemical and physical properties offer an outstanding platform for material and biological applications. The operationalization ability and colloidal stability of gold nanoparticles help researchers to exploit and apply them in various theragnostic applications [84]. The genotyping of the subgroups of frequently occurring thalassemia mutations, including α-thalassemia 1 (SEA and THAI deletion) and α-thalassemia 2 (3.7-kb and 4.2-kb deletion) were performed by Chomean et al. through the development of a novel calorimetric nanogold probe. The approach involved a two-step hybridization process, where the nanogold mixed probes were hybridized with the target DNA in the first step, which proceeded depending on the color changes. The blue color indicated no abnormal genes, whereas, a purple or red color indicated the presence of abnormal genes, hinting that the samples should be further processed with nanogold single probes. The consistency of the results, with respect to the standard agarose gel electrophoresis, demonstrated the accuracy, precision, sensitivity, specificity, simplicity, and field applicability of this approach, making it a valid option to be employed for other genetic studies [85].

Doria et al. developed gold nanoparticle-based systems to detect b-globin gene variants. This simple, efficient, and cost-effective assay was specifically designed to detect particular DNA/RNA sequences based on the non-crosslinking hybridization method. A higher sensitivity had been achieved by integrating this method on a nanocrystalline silicon device and the three commonly occurring beta-globin gene mutations: IVS1 and nt1(G→A); IVS1 and nt2 (T→C); IVS1 and nt6 (T→C), causing thalassemia were efficiently detected [86].

The combination of ligase detection reaction and PCR with a unique nanogold-based universal array for the detection of various point mutations present in fetal DNA from maternal plasma samples was investigated by Yi et al. Following the sensitivity and specificity analysis, a low abundance specific mutation, IVS2 654(C→T), in the β-globin gene in thirty maternal plasma samples was found through this assay. A high accuracy in the developed method was demonstrated by obtaining the same results in these samples by performing PCR/reverse dot blot of amniotic fluid cell DNA [87]. In another study, Gholivand et al. developed an electrochemical genosensor based on Au nanoparticles-poly (4-aminothiophenol)/reduced graphene oxide/glassy carbon electrode (AuNPs-PAT/rGO/ GCE). This sensor had a higher accuracy when detecting β-thalassemia genes and functioned on the principle of immobilization and hybridization of a thiol-tagged oligonucleotide probe with the target sequence [88].

Han et al. also succeeded in developing an electrochemical biosensor based on electrodes modified by ferrocenoyl cysteine conjugates, to detect globin gene variants in diseased conditions. The efficient quantitative analysis of hemoglobin was conducted by adsorbing the electroactive materials into the gold nanoparticles [89]. The DNA-based piezoelectric biosensors were prepared using the immobilization of oligonucleotide probes at the gold electrodes and were used for the detection of the β-thalassemia mutation C→T substitution in codon 39 of the beta-globin gene [90].

#### 4.3.2. Silver Nanoparticles-Based Diagnosis

Silver nanoparticles with higher stability and the in vitro detection of ultra-sensitive molecules made them efficacious in the development of novel biosensors for the detection of hemoglobin. Ye et al. developed molecularly imprinted polymers modified by Ag nanoparticles (NPs)/PbTiO3 electrodes to detect and quantify hemoglobin [91]. The nano-based detection of the α-thalassemia 1 mutation (SEA deletion) was demonstrated in, yet, another study whereby quartz crystal microbalance (QCM) was developed for the identification of the abnormal gene, using the silver electrode immobilized with a biotinylated probe on the QCM surface. The diagnostic test, using silver thalassemic QCM, proved to be specific, sensitive, rapid, cheap, and field applicable and valid in performing a one-step diagnosis of α-thalassemia1, without the need for a preliminary screening test [92].

### 4.4. Other Nano-Diagnostic Approaches

The electrospun nanofibers (NFs) have been used to intercalate the unique dye-intercalated DNA dendrimer probe (G3SG) and to develop a platform for amplified fluorescent sensing for the detection of nucleic acids, proteins, and cancer cells. The large surface area to volume ratio of the nanofibers and strong emission intensity of dendrimer probes have allowed the detection of a 20 pM of thalassemia causing mutated beta-globin gene fragment, and thrombin and HeLa cells with high sensitivity and selectivity [93].

The fluorescein-containing probe-gated mesoporous silica nanoparticles (MCM-41) were used to design a genotyping assay to detect the thalassemia causing mutation IVS110 (A > G reversion). The hybridization of the mutated or wildtype probe nucleotide sequence with a single-stranded target DNA sequence was performed by entrapping the fluorescein molecules in the pores. The mutated targets provided different fluorescent signals, which helped in the detection of the mutant samples [94].

Hemoglobin in anemic pregnant women was quantified in a study using the development of NiTe nanorods-based non-enzymatic sensors and demonstrated the use of the nanorods in an electrochemical analysis of hemoglobin in thalassemic patients [95]. Dolak et al. successfully synthesized a molecularly imprinted cryogel based on lanthanide-chelate, in accordance with the cryopolymerization techniques, for the selective separation of hemoglobin from serum, demonstrating a 94.34% recovery, thus, could successfully be used in the diagnosis of thalassemia [96].

**Table 2:** Nanotechnology-based diagnostic approaches for thalassemia.

Nanoparticles	Modification	Application	Method	Detection Limit	Reference
**Carbon**	Co_3_O_4_ doped carbon nanofiber (CNF) composite modified on carbon ionic liquid electrode (CILE)	Detection of electrochemical, conformational, and structural alterations of hemoglobin	Immobilization of hemoglobin on the biosensor	0.1 mmol L^−1^	[75]
	Carbon paste-based electrode modified with 1-ethyl-3-methylimidazolium chloride and CdO-nanoparticle	Determination of deferoxamine and vitamin C in the thalassemic patients	Multivariate curve resolution alternating least (MCR-ALS) algorithm	0.030 μM	[76]
**Graphene**	Palladium–graphene (Pd–GR) nanocomposite-modified carbon ionic liquid electrode	Fabrication of third-generation electrochemical biosensor for the detection of hemoglobin	Fixing Hb to Pd–GR nanocomposite	0.35 mmol L^−1^	[78]
	3D porous hybrid of graphitic C_3_N_4_ nanoparticle decorated in the assembly of graphene and Co_2_Al layered double hydroxide nanosheets	Production of third-generation biosensor	X-ray diffraction, electron microscopy, X-ray photoelectron spectroscopy	0.05 mM	[79]
	Graphene oxide-tellurium nanowires (TeNWs/GO)	Quantitative determination of hemoglobin β-thalassemia major patients	Detection of electrical response by redox reaction due to electrical stimulus to the biochemical system	0.29 μM	[70]
**Quantum Dot**	Coupling of quantum dots with magnetic nanoparticle-based probes	Detection of point mutation in the human beta-globin gene (IVS-II-I G → A point mutation)	Ligase reaction proceeded with the allele-specific probes	-	[81,82]
	CdS/TiO_2_ nanocomposite-based molecularly imprinted photo-electrochemical sensor	Detection of hemoglobin under visible light irradiation	Principle of decrease in the photocurrent in the case of attachment of hemoglobin to the sensor	0.53 pg/mL	[83]
**Gold**	Calorimetric nanogold probe	Genotyping of subgroups of α-thalassemia 1 and α-thalassemia 2	Two-step hybridization of target DNA with nanogold mixed probes and nanogold single probes	-	[85]
	Gold nanoparticle-based systems integrated with nanocrystalline silicon device	Detection of mutation in the b-globin gene	Non-crosslinking hybridization	-	[86]
	Nanogold-based universal array	Detection of point mutations from fetal DNA in maternal plasma samples	PCR and ligase detection reaction	-	[87]
	Thiol-tagged oligonucleotide probes on the Au nanoparticles- (AuNPs-PAT/rGO/GCE)	Detection of β-thalassemia gene	Hybridization of oligonucleotide with the target sequence	-	[88]
	ferrocenoyl cysteine conjugates adsorbed onto gold nanoparticles	Quantitative analysis of hemoglobin	Adsorption	0.03 μg/mL	[89]
	Piezoelectric biosensors based on gold electrodes	Detection of β-thalassemia mutation C→T substitution in the codon 39 of the HBB gene	Immobilization of oligonucleotide probes on the electrodes	-	[90]
**Silver**	Molecularly imprinted polymers modified by Ag nanoparticles (NPs)/PbTiO_3_ electrodes	Detection and quantification of hemoglobin	-	0.23 pM	[91]
	Silver electrode coupled with Quartz Crystal Microbalance (QCM)	Identification of thalassemia gene mutations	Immobilization of biotinylated probe on the QCM surface using silver electrode	0.5 μmol/L	[92]
**Dendrimer**	Dendrimer probe (G3SG) intercalated with electrospun nanofibers	Detection of β-thalassemia gene fragments	Amplified fluorescent sensing	20 pM	[93]
**Mesopores**	Mesoporous silica nanoparticles (MCM-41) loaded with reporter fluorescein molecules	Detect of thalassemia causing mutation IVS110 (A > G reversion)	Genotyping assay	-	[94]
**Nickel**	NiTe nanorods	Electrochemical analysis of hemoglobin in thalassemic patients	Non-enzymatic sensor-based quantification	0.012 nM	[95]
**Cryogel**	Molecularly-imprinted cryogel based on lanthanide-chelate	Diagnosis of thalassemia	Cryopolymerization techniques for selective separation of hemoglobin from serum	-	[96]

## 5. Nanotechnology for the Treatment of Complications of Thalassemia

**Figure 3:**
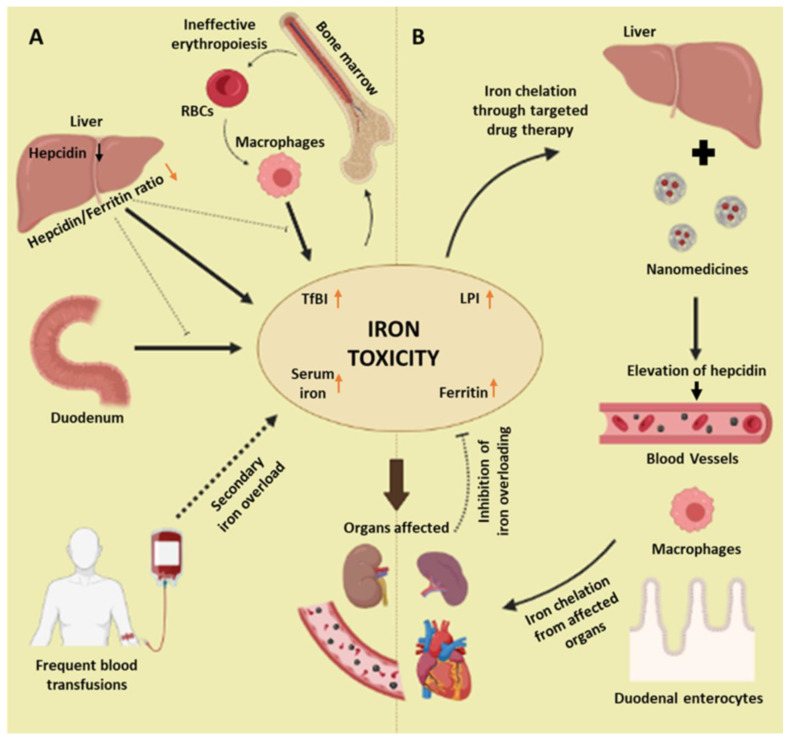
Proposed mechanism of targeted drug therapy using nanomedicines. (**A**) Iron toxicity, which includes the increase in the serum iron level, ferritin, transferrin bound iron, and labile plasma iron occurs due to frequent blood transfusions and genetic defects in thalassemia patients. Hepcidin, which is the major iron metabolism machinery produced in the liver, decreases drastically, thus, decreasing the hepcidin to iron ratio, which causes the duodenal enterocytes, hepatocytes, and macrophages to not function properly, resulting in further elevations in the iron levels. This causes ineffective erythropoiesis and abnormal production of RBCs along with iron accumulation in various organs. (**B**) To overcome iron toxicity, targeted drug therapy using nanomedicines is performed to chelate the iron. The nanomedicines will target the hepcidin-producing cells in the liver, thus, elevating the hepcidin level to reduce the iron levels in the affected organs, via the correct functioning of vessels, macrophages, enterocytes, and hepatocytes. (TfBI: transferrin-bound iron; LPI: labile plasma iron).

In thalassemia patients, hemoglobinopathy-related complications, specifically anemia, need to be overcome. This is usually achieved by frequent blood transfusions, which cause secondary iron overload [97]. The iron homeostasis in the body is maintained by the hepcidin–ferroportin conjunction in the liver. Ferroportin, which is an iron export protein, is regulated by hepcidin, which is a hormone produced in the liver. However, in thalassemic patients, the dysregulation of iron metabolism causes the suppression of hepcidin and results in a marked decrease in the hepcidin-to-ferritin ratio. This iron toxicity due to ineffective erythropoiesis, hepcidin suppression, and frequent transfusions causes damage to the vital organs and, ultimately, the death of the patients (Figure 3) [98,99,100,101].

Conventional iron chelation therapies involving three successfully developed and FDA-approved drugs i.e., deferoxamine, deferiprone, and deferasirox are being widely used to reduce the toxic iron levels in these patients. However, considering the mortality rate due to iron toxicity, the adverse effects of conventional drugs on ocular, auditory, and renal pathways, and the increasing toxicity at high or prolonged dosages, more robust and efficient therapeutic methods are needed for these patients [102,103,104,105]. Nanoparticles offer a diversity of therapeutic advantages in the treatment and modulation of several genetic and immunogenic disorders [106]. It is a rapidly emerging field focused on overcoming the hindrances associated with conventional drug delivery systems and other therapeutic regimes [107]. Researchers are trying to use nanoparticles for therapeutic purposes, including targeted drug delivery, gene delivery, and gene editing to eliminate the disorder, as indicated in Table 3 [108,109]. The researchers have made efforts to develop artificial hemoglobin based on nanotechnological advancements to overcome the burdensome transfusion therapies. This has been achieved by integrating hemoglobin enzymes, such as catalase and superoxide dismutase into a nanocomplex to produce biodegradable polymeric membranous artificial RBCs, which may act as oxygen carriers as well as an antioxidant depending on the conditions [110].

Reduction in hepcidin expression due to *HAMP* downregulation and *Tmprss6* upregulation may cause significant increases in iron levels [111,112]. Studies conducted on mice models indicated the use of antisense oligonucleotides to pharmacologically reduce *Tmprss6* expression and elevate *HAMP* expression to increase hepcidin production and decrease toxic iron levels [112,113,114]. This concept was utilized to develop lipid nanoparticle (LNP)–formulated small interfering RNAs (siRNAs) in th3/+ mice, to produce a complex of *Tmprss6* inhibitors with nano-based iron chelators [114,115]. This has proven to be successful in reducing iron overload and ineffective erythropoiesis in not only thalassemia patients but in hemochromatosis and other hemoglobinopathies [111,116].

The comparison of the effectiveness of a novel nanochelator, TLc-A based on nanochelating technology and the conventional iron-chelating agent, deferoxamine was conducted using both in vivo and in vitro studies. TLc-A reduced iron overload more effectively in both the Caco2 cell line and iron-intoxicated rats, indicating the higher efficiency of nanochelating agents [117]. However, the use of nanochelating agents might work differently in different individuals, as indicated in another study investigating the efficacy of graphene oxide nanoparticles, along with the acquired protein corona, during the treatment regimen, and hinted towards the efficient use of personalized medicine, according to the requirement and immune-mediated reactions of the individuals [118].

Ali et al. investigated the interaction of nickel–zinc–iron oxide (Ni_0.5_Zn_0.5_Fe_2_O_4_) and cobalt ferrite (CoFe_2_O_4_) with human erythrocytes, with respect to their hemolytic activity. Moreover, their effect on the albumin in the plasma of β–thalassemia major patients. The interaction of these nanoparticles induced morphological changes in the erythrocytes. Ni–Zn ferrite nanoparticles were found to decrease hemolysis in thalassemia patients, compared to cobalt ferrite nanoparticles, which were found to increase hemolysis in the patients. The authors suggested the possible reason for the differences in the function of the nanoparticles as their tiny size and unique physiochemical properties demonstrating the Ni–Zn ferrite was more efficacious in thalassemia treatment compared to the cobalt ferrite nanoparticle [119]. The efficient use of silver nanoparticles modified by the tannin fraction of Myrtus communis extract (MC-AgNPs) to chelate iron in a thalassemic rat in vivo model was demonstrated and revealed a satisfying effectiveness at lowering the excess iron [120].

Ergün et al. prepared Fe^3+^ imprinted beads embedded with cryogels, which were used to chelate iron from the plasma of β–thalassemia patients effectively [121]. The efficacy of mesoporous silica nanoparticles with EDTA and amine groups in the pores and prepared by co-condensation reaction was investigated in vivo and in vitro and, in both, were demonstrated to be effective for iron chelation in thalassemic patients [122]. Polyrotaxane-based nano chelators (rPR-DFO) have been prepared and tested in vivo using mice models to remove excessive systematic and hepatic iron in thalassemic conditions, which has proven to be a promising system for iron chelation therapy [123]. The clinically used chelator, desferrioxamine-loaded polymeric nanoparticles (NPs) containing galactose as the targeting ligand were prepared in another study for targeted drug delivery to asialoglycoprotein receptors in hepatocytes [124]. Zinc oxide nanocrystals–methylene blue nanocomposites [125], pyrophosphate functionalized silver nanoparticles (Pyro-AgNPs) [126], gold nanorods [127], and HRP–AuNP–CaCO_3_ composites [128] have been developed to sense deferiprone in vitro, which is an anti-thalassemic iron chelating drug.

Capretto et al. used microfluidic technology to produce polymeric micelles encapsulating the DNA-binding drug mithramycin (PM-MTH), which had improved controllability, reproducibility, smaller size, lower toxicity, and polydispersity. It could upregulate γ-globin expression, thus, increasing HbF content and alleviating the symptoms associated with β-thalassemia [129]. The efficient γ-globin expression in the hematopoietic stem cells was also achieved by the development of episomal vectors based on the scaffold/matrix attachment region (S/MAR) for episomal retention and the β-globin replicator [130]. This method was investigated both in mouse models and human hematopoietic stem cells provided efficient results and could be employed to enhance the fetal hemoglobin production in thalassemia patients to ameliorate the clinical complexities. Recently, gene editing technology, CRISPR/Cas9, in conjunction with a supramolecular nanosubstrate–mediated delivery (SNSMD) strategy has been used to knockout the defective HBB gene in various hemoglobinopathies, while the in vivo proliferative efficacy was also observed in mice with sickle cell. The positive outcome demonstrated that this method could be employed for the treatment of other hemoglobinopathies as well as thalassemia [131].

**Table 3:** Nanotechnology-based therapeutic applications for thalassemia patients.

Nanoparticles	Use	Reference
Enzyme-integrated polymeric membrane	Production of artificial hemoglobin	[110]
Deferoxamine-loaded polymeric nanoparticles	Targeted drug delivery	[124]
Mithramycin-encoded polymeric micelles (PM-MTH)	Upregulation of γ-globin expression to increase HbF content	[129]
Nickel–zinc–iron oxide	Decrease the hemolysis in thalassemia patients	[119]
Lipid nanoparticle (LNP)-formulated small interfering RNAs (siRNAs), TLc-A based nanochelator, graphene oxide, MC-AgNPs, cryogel, mesoporous silica nanoparticles, polyrotaxane-based nanochelator	Removal of excess iron from the plasma of β–thalassemia patients	[111,112,113,114,115,116,117,118,120,121,122,123]
ZnO nanocrystals–methylene blue nanocomposites, pyro-AgNPs Au nanorods, HRP–AuNP–CaCO_3_ composites	In vitro sensing of anti-thalassemic iron chelating drug i.e., deferiprone	[125,126,127,128]
Supramolecular nano substrate–mediated delivery (SNSMD)	Knockout of the defective HBB gene using CRISPR/Cas9	[131]

## 6. Conclusions and Future Perspectives

The therapeutic and diagnostic regimen of hemoglobinopathies, specifically thalassemia has always been challenging. The genetic mutations, clinical manifestations, and therapies vary among different populations and individuals. The conventional theragnostic approaches are being used to lessen the physical and mental constraints on these individuals. However, with technological advancements and with the increasing recognition of nanotechnology, nanoparticles, and nanomedicines are being successfully used in the diagnosis of various diseases, disease mutations, and therapeutic efficiencies of conventional methods. They have also been employed for targeted drug delivery, gene therapy, and gene editing. The development of biosensors using various nanoparticles has proven to be highly efficacious, simple, time, and cost-effective in the diagnosis of thalassemia. The biosensors using different nanoparticles employ various electrochemical and hybridization techniques, which may help in the effective diagnosis of different mutations involved in the pathogenesis of thalassemia and can also overcome the cost and time constraints. Many nanoparticles have been investigated for their therapeutic efficiencies for producing artificial hemoglobin, as nanochelating agents, and as nanocarriers to be used in conjunction with gene therapy and gene editing technologies. Depending on the size and properties of various nanoparticles, iron chelation in thalassemia patients, along with HbF induction, a decrease in hemolysis, and the knockout of defective genes have been successfully achieved. In view of the efficacy, complexity, and cost of current diagnosis and treatment methods, new, simplified, advanced, and cost-effective methods are required to reduce the physical and psychological burden on these patients. Based on these diagnostic and therapeutic approaches more efficient and cost-effective methods could be developed to detect thalassemia mutations and treat the patients effectively. The efficacious diagnosis and the useful treatment criteria may help in the development of nano-based personalized medicines, which could provide a breakthrough in the theragnostic criteria of thalassemia and other hemoglobinopathies.

## Figures and Tables

**Table 1:** The conventional therapeutic approaches in use along with their efficacy in the amelioration of the adverse symptoms and treatment of thalassemia.

Treatments	Efficacy	Disadvantages	References
Transfusion therapy	Overcomes anemic condition	Iron overload, expensive	[2,5,12,23,25,28,31,32]
Iron chelation therapy	Maintains body iron at safe levels.	Disrupted physiological conditions, blurred vision, rashes	[12,21,23,25,28,31,32]
HbF induction through hydroxyurea, DNA methylation inhibitors, and short-chain fatty acids	Increases γ-globin production to upregulate total hemoglobin levels, ameliorate anemia, and diminishes phosphatidylserine expression on RBCs.	Ulcers, organ damage, breathing problems, skeletal deformities	[2,12,25,27,28,29,32]
Ineffective erythropoiesis signaling modulators	Increases hemoglobin in a dose-dependent fashion by targeting JAK2/STAT5 signaling pathway	Physiological complications, expensive	[27]
Bone marrow transplantation	Restore the tissue’s capability of synthesizing functional hemoglobin	Graft-versus-host disease, cataract, organ damage, physiological complications	[31,32]
Hematopoietic stem cell transplantation	Reduces intensity or non-myeloablative conditioning and limits iron burden, and comorbidities	Decreased immunity, infections, graft-versus-host disease, death	[2,21,23,25,28,32,33]
Splenectomy	Alleviate anemia in non–transfusion-dependent thalassemia, less effective	Infections, sepsis, increased bleeding, injured organs	[2,21,23,31]
Gene therapy	Regulates globin genes expression through locus control region and promoter region, may include beta globin replacement or fetal globin reactivation	Genotoxicity, allergic reactions, increased risk of cancer, expensive	[12,22,23,25,28,29,31,32,33]
Gene editing	Allow the sustained production and endogenous regulation of the globin proteins by targeting the BCL11A gene, including zinc-finger nucleases (ZFN), transcription activator-like effector nucleases (TALENS), and clustered regularly interspaced short palindromic repeats (CRISPR) and CRISPR-associated-nuclease 9 (CRISPR-Cas9) as gene editing tools	Insufficient transduction efficiency, dysregulated transgene expression, increased risk of gene silencing	[12,22,24,29,31]

## Data Availability

Not applicable.
